# Strain Rate and Temperature Influence on Micromechanisms of Plastic Deformation of Polyethylenes Investigated by Positron Annihilation Lifetime Spectroscopy

**DOI:** 10.3390/polym16030420

**Published:** 2024-02-02

**Authors:** Cezary Makarewicz, Marta Safandowska, Rafal Idczak, Slawomir Kolodziej, Artur Rozanski

**Affiliations:** 1 Centre of Molecular and Macromolecular Studies, Polish Academy of Sciences, Sienkiewicza 112, 90-363 Lodz, Poland; cezary.makarewicz@cbmm.lodz.pl (C.M.); marta.safandowska@cbmm.lodz.pl (M.S.); 2 The Bio-Med-Chem Doctoral School of the University of Lodz and Lodz Institutes of the Polish Academy of Sciences, Banacha 12/16, 90-237 Lodz, Poland; 3 Institute of Experimental Physics, University of Wroclaw, pl. Maksa Borna 9, 50-204 Wroclaw, Poland; ridczak@ifd.uni.wroc.pl; 4 Institute of Materials Science, University of Silesia in Katowice, 75 Pulku Piechoty 1A, 41-500 Chorzow, Poland; slawomir.kolodziej@us.edu.pl

**Keywords:** semicrystalline polymers, plastic deformation, cavitation, positron annihilation lifetime spectroscopy (PALS)

## Abstract

Plastic deformation of low/high density polyethylene (LDPE/HDPE) was analyzed in this work using positron annihilation lifetime spectroscopy (PALS). It was shown that in undeformed LDPE, both the mean ortho-positronium lifetime (τ_3_) and its dispersion (σ_3_), corresponding to the average size and size distribution of the free-volume pores of the amorphous component, respectively, were clearly higher than in HDPE. This effect was induced by a lower and less uniform molecular packing of the amorphous regions in LDPE. During the deformation of LDPE, an increase in the τ_3_ value was observed within the local strains of 0–0.25. This effect was mainly stimulated by a positive relative increase in interlamellar distances due to the deformation of lamellar crystals oriented perpendicular (increased by 31.8%) and parallel (decreased by 10.1%) to the deformation directions. At the same time, the dimension of free-volume pores became more uniform, which was manifested by a decrease in the σ_3_ value. No significant effect of temperature or strain rate on the τ_3_ and σ_3_ values was observed during LDPE deformation. In turn, in the case of HDPE, with an increase in the strain rate/or a decrease in temperature, an intensification of the cavitation phenomenon could be observed with a simultaneous decrease in the τ_3_ value. This effect was caused by the lack of annihilation of ortho-positonium (o-Ps) along the longer axis of the highly anisotropic/ellipsoidal cavities. Therefore, this dimension was not detectable by the PALS technique. At the same time, the increase in the dimension of the shorter axis of the cavities was effectively limited by the thickness of amorphous layers. As the strain rate increased or the temperature decreased, the σ_3_ value during HDPE deformation increased. This change was correlated with the initiation and intensification of the cavitation phenomenon. Based on the mechanical response of samples with a similar yield stress, it was also proven that the susceptibility of the amorphous regions of LDPE to the formation of cavities is lower than in the case of amorphous component of HDPE.

## 1. Introduction

Semicrystalline polymers, characterized by a complex, multilevel structure, exhibit unique physical properties that allow them to replace other types of materials, such as wood, glass or steel, in various industrial applications [1]. The advantages of these materials led to their use in numerous commercial applications, including pipes [2,3], packaging [4,5] automotive parts [6], electrical appliances [7] and many others. Moreover, blends of semicrystalline polymers are also used to develop materials with balanced or improved mechanical, thermal or barrier properties [8,9,10]. Considering the wide range of applications of semicrystalline polymers, it is also essential to know their properties under various conditions, including different temperatures or strain rates.

It is well known that semicrystalline polymers typically crystallize during solidification, resulting in a structure consisting of stacked lamellar crystals and amorphous layers. The amorphous regions are characterized by local heterogeneity as they contain fragments of macromolecules forming entanglements, tie-molecules, chains ends and other non-crystallizable units (chain branches) or substances (oligomers, stabilizers) [11]. Due to their structure, plastic deformation of semicrystalline polymer is a complicated process based on the cooperative and simultaneous responses of crystalline and amorphous components. Recently, we have shown that the elastic modulus of the amorphous phase of semicrystalline polymers (above the glass transition temperature) is two orders of magnitude lower than that of lamellar crystals [12,13]. Thus, the initial stage of deformation of semicrystalline polymer takes place mainly in the interlamellar regions due to the significantly lower value of the critical stress required to activate deformation of the amorphous phase compared to the crystalline phase [14]. Crystals characterized by greater “rigidity” play a passive part in the deformation of the amorphous component, acting as non-deformable objects. Measurable deformation of the amorphous component appears before the macroscopic yield point is reached and takes place according to three different mechanisms, depending on the orientation of lamellae in relation to the deformation direction. These mechanisms are: separation of lamellae, interlamellar slips and rotation of lamellar crystals stacks [15,16,17].

In the literature, two different mechanisms of plastic deformation of crystalline component have been proposed. Peterson described this phenomenon as the emission of dislocation from the edges of the lamellar crystals and their subsequent movement due to the crystallographic slips. This concept was further explored by Shadrake and Guiu [18,19,20]. Two kinds of crystallographic slips can be distinguished: fine and coarse. Both of them occur in planes containing chains as a result of the generation and progression of dislocations. Coarse slips cause the formation of block structures from continuous lamellae, leading to lamellae fragmentation [21,22]. In contrast, fine slips are responsible for changing the angle between the chain and the normal to the plane of the lamellae, leading to lamellae rotation and thinning. The second mechanism of the plastic yielding of semicrystalline polymers is associated with non-crystallographic changes of the initial crystalline skeleton. It has been demonstrated that adiabatic heating results in partial melting and recrystallization, leading to the formation of new crystalline structures [23,24,25].

During the deformation process of semicrystalline polymers in a stretching mode, a phenomenon called cavitation is usually observed. The appearance of cavities leads to whitening of the polymeric material when the size of cavities is comparable to the wavelengths of visible light [26]. Around the yield point, the plastic deformation of crystals is a process that competes with cavitation phenomenon. Its presence is dependent on the microstructure of a material and the deformation conditions. In general, cavitation occurs when crystals have higher plastic resistance (thicker lamellae), whereas in the case of crystals with lower plastic resistance (thinner lamellae), plastic deformation of the crystalline component is observed without cavitation [7,27,28,29,30,31]. Cavitation is one of the main reasons for a volume increase during the deformation process. However, the overall value for the volume variation is the sum of three components: elastic, plastic and cavitational [25]. Due to the lack of internal structure, cavities are unable to transfer stress, unlike crazes that also appear in semicrystalline polymers [21].

The structure of the amorphous phase has a strong influence on cavitation. The intensity of cavitation was found to be higher for polymeric materials with a lower molecular weight or a reduced density of entanglements [32,33]. The presence and intensity of cavitation is dependent on the density of stress transmitters in the interlamellar regions, which was proved by Humbert et al. [34]. Significant changes in the cavitation phenomenon can also be achieved through the modification of interlamellar regions at the free-volume level [35,36,37]. In our recent studies, we removed potential cavitation-nucleation sites using supercritical CO_2_ extraction. This process did not alter the crystalline skeleton of the material but, surprisingly, it intensified the cavitation phenomenon. On the other hand, the presence of an appropriate modifier in the amorphous-phase regions can eliminate the cavitation phenomenon. An increase/decrease in the content and size of free-volume pores in the amorphous regions of purified/modified samples was the reason for the observed effects. These studies proved that the mechanism of cavitation has a homogeneous nature, and that it is based on local fluctuations in free volume within amorphous regions.

Experimental conditions (temperature, strain rate) likewise have a strong influence on the micromechanisms activated during the deformation process. The appearance of the cavitation phenomenon depends upon the deformation temperature. First of all, it can only be observed above the glass-transition temperature [21]. Xiong et al. mapped the initiation of three processes: crystal shear, martensitic transformation and cavitation for polyethylene as a function of the deformation temperature. It was proved that the occurrence of cavitation is delayed when the temperature of deformation is increasing [38]. For most semicrystalline polymers, stretching at an elevated temperature results in a decrease in tensile stress and an increase in deformability [39]. Merah et al. investigated the tensile properties of high density polyethylene (HDPE) in the temperature range from −10°C to 70°C [40]. In presented studies, they demonstrated that the yield stress and elastic modulus decrease linearly with an increase in deformation temperature. Enikolopian et al. studied the plastic deformation of low-density polyethylene (LDPE) of a temperature increase from 20 °C to 105 °C (melting point) [39]. It was proven that an increase in temperature causes an increase in the inhomogeneity of the microstructure due to the gradual destruction (melting) of less organized and imperfect crystals.

It is also well known that a higher strain rate usually leads to an intensification of the cavitation phenomenon, a higher value of yield stress and earlier sample fracture [41,42]. Dijkstra et al. noted that at high strain rates, the shape of the stress-strain curve significantly changes. [43] A sharp downward slope appears after reaching maximum stress and the stress plateau disappears. This effect corresponds with the studies of Cessna, which concluded that a transition in deformation behavior due to an increase in strain rate is accompanied by a gradual increase in volume strain [43,44]. Hobeika et al. also proved that increasing a strain rate increased the volume fraction of sheared crystalline component due to an increase in slipping surface area [45]. Pawlak et al. concluded that during slower deformation crystals are more able to undergo a plastic deformation process [42]. Due to the easier deformation of crystals under slow strain rates, the cavitation phenomenon may not occur or may be unstable (healing process).

The Small Angle X-ray Scattering (SAXS) technique is commonly used to investigate deformation-induced changes in the microstructure of semicrystalline polymers [46]. However, the very high electron-density contrast between cavities and the polymer matrix enables the observation of microstructure evolution within cavitating material during tensile drawing [47]. Additionally, the typical setup configuration for SAXS measurements only allows for the observation of objects with sizes ranging from several nanometers to several dozens of nanometers [48]. Humbert et al. studied cavitation evolution during the deformation of polyethylene using in situ SAXS measurements [34]. The smallest dimension of cavities recorded in this research was around 40 nm (at nominal strain around 35%), which was significantly higher than value of long period (22 nm). They concluded that the very first stage of the nucleation process evolves quickly from 0 to 40 nm and cannot be recorded by SAXS. In our previous work [49] dedicated to the analysis of the plastic deformation of HDPE, the minimum radius of gyration of the cavities, as determined by SAXS measurements, was 16.9 nm (under nominal strain around 35%). 

Positron Annihilation Lifetime Spectroscopy (PALS) is one of the most valuable techniques for investigating the size and distribution of free-volume pores. It is used to study the microstructure of metals, ceramics and polymeric systems [50]. In this technique, a positron generated by a radioactive source is implanted into a solid sample and subsequently annihilated with a random electron. This process is called bulk/free annihilation, and it occurs very fast because the average positron lifetime is around 100–500 ps. During the material investigation using the PALS technique, some of the positrons injected into the solid can form a state called positronium (Ps). Ps can exist in two spin states: para-positonium (p-Ps) and ortho-positonium (o-Ps), in a ratio of 1:3. In a vacuum, the lifetime for p-Ps is 0.125 ns, while it is 142 ns for o-Ps. However, when o-Ps is formed inside solid matter it becomes sensitive to the size of free-volume pores within the investigated material, and its lifetime typically decreases to 1–10 ns [51].

**Figure 1:**
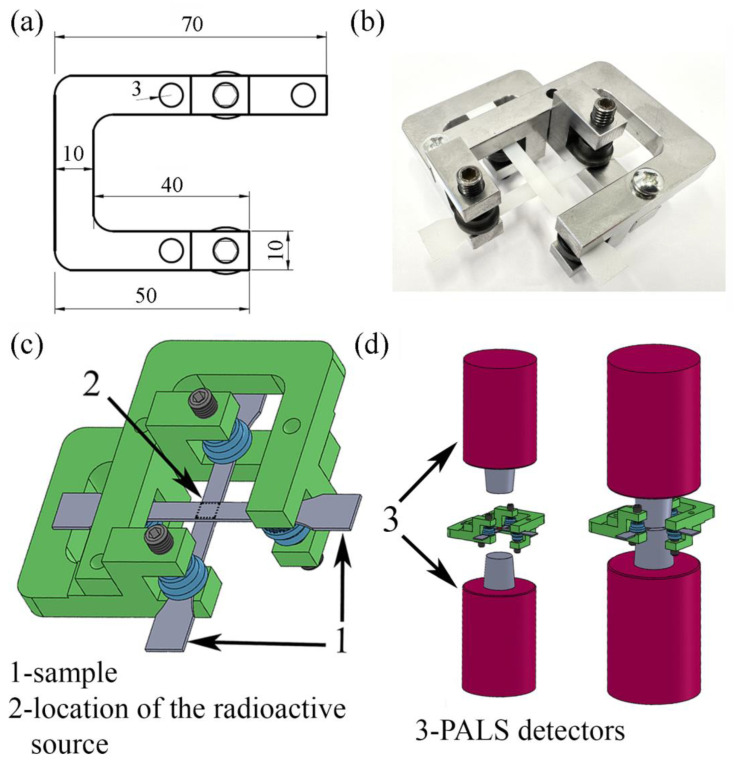
Technical (**a**) and real (**b**) visualization of the frame system; 3D visualization of frames system holding deformed samples during PALS measurement (**c**); location of frames with samples between PALS detectors (**d**) [49].

Using PALS measurement, one can determine the size of empty spaces within the range of 0.25–16 nm. However, due to the long exposure time of the sample during a single PALS measurement, an in situ analysis of the tensile deformation process was impossible. Therefore, in our previous papers, we described an alternative method for analysis of the deformation process using the PALS technique, which is treated as a pseudo-in situ analysis [49,52]. In this method, two samples deformed to the same value of local strain (LS) were securely fixed in special frames to prevent relaxation processes (Figure 1). The radioactive source was placed between these samples and the whole set was assembled between PALS detectors. For each experiment, new samples were stretched to the selected strain values and fixed in the frames for the collection of PALS data. Thus, this method allows for the investigation of changes in the size and distribution of free-volume pores across a desired range of strains, similar to in situ tests [49,52]. Recently, a new spectrometer with a high count rate was described as an alternative setup for in situ PALS measurements [53].

Most of the studies on the deformation of semicrystalline polymers are focused at or significantly above the yield-point strain values. Due to this fact, there is still a lack of knowledge about the initial stages of deformation-induced changes in microstructure of semicrystalline polymers. Therefore, in this study, the evolution of size and the distribution of free-volume pores within amorphous regions up to the yield point was investigated over a wide range of strain rates and temperatures during the uniaxial deformation of two semicrystalline polymers—HDPE and LDPE. The influences of drawing conditions on the micromechanisms of deformation activated in both polymers were analyzed mainly using the PALS technique.

## 2. Experimental Section

### 2.1. Materials

Studies were performed on two raw materials produced by Lyondell Basell (Houston, TX, USA): high density polyethylene (HDPE) Hostalen GC 7260 with MFI = 8 g/10 min (at 190 °C, 2.16 kg according to ISO 1133 [54]), and low-density polyethylene (LDPE) Lupolen 1840 D with MFI = 0.25 g/10 min (at 190 °C, 2.16 kg according to ISO 1133).

### 2.2. Sample Preparation

Samples for all measurements were cut from 1 mm thick films prepared by compression molding (5 min, 50 MPa) from raw material using a hydraulic press (at 180 °C). Samples were solidified between metal plates.

### 2.3. Mechanical Testing

Mechanical properties of polymers were assessed with the use of a Instron 5582 tensile testing machine produced by Instron (Norwood, MA, USA) within a load range 0–2 kN for tests performed at room temperature and within 0–100 kN for tests performed in temperature-controlled chamber Instron 3119-606. The dumbbell-shaped samples were prepared according to ISO 527-2 standard with 1 mm thickness, 5 mm width and 25 mm gauge length [55]. HDPE was stretched at room temperature with three strain rates: 3.3 × 10^−2^ s^−1^, 3.3 × 10^−3^ s^−1^ and 3.3 × 10^−4^ s^−1^ (with speeds of crosshead 50 mm/min, 5 mm/min, 0.5 mm/min, respectively) and with selected strain rate: 3.3 × 10^−3^ s^−1^ at −30 °C and 70° C. LDPE-stretching tests were performed at room temperature with strain 3.3 × 10^−3^ s^−1^, and at temperature −30 °C with strain 3.3 × 10^−2^ s^−1^. To estimate LS and volume strain, dot markers were placed along the gauge region of the samples. LS was calculated as a change in distance between the dot markers according to the equation (l − l_0_)/l_0_, where l_0_ is a distance between markers for the undeformed sample and l is a distance between markers for the deformed sample. Details about methodology and calculations have been described in other papers [36,56,57]. In addition, the actual three dimensions of a deformed samples were recorded by a video camera and used for the precise determination of true strain/stress values.

### 2.4. Differential Scanning Calorimetry (DSC)

Thermal analysis of samples was conducted using an indium-calibrated calorimeter Q 20 produced by TA Instruments (New Castle, DE, USA). Melting endotherms were collected during the heating process with a heat rate of 10 °C/min under nitrogen flow. The crystallinity degree was estimated using the equation:

(1) $$X_{c}=\frac{∆H_{m}}{∆H_{m}^{0}}$$

where ΔH_m_ is the experimental enthalpy of the sample and $∆H_{m}^{0}$ is enthalpy of HDPE or LDPE crystal having infinite crystal thickness.

### 2.5. Small-Angle X-ray Scattering (SAXS)

Two-dimensional small-angle X-ray scattering (2D SAXS) measurements were performed using a 2D SAXS diffractometer to detect cavities and analyze the microstructure of samples. The scattering measurements were performed using the setup described in previous papers [49,58]. Additionally, in situ measurements of the deformation process were made with the use of a synchrotron radiation beamline as described elsewhere [41]. The values of long period were measured according to the procedure presented in previous papers [35,56,57,59].

### 2.6. Positron Annihilation Lifetime Spectroscopy (PALS)

Positron lifetime spectra were collected in air at room temperature with the use of a “fast-fast” ORTEC spectrometer, which has a resolution of 300 ps. In this technique, positrons emitted from the ^22^Na radioactive source, which had been formed into a disc with a diameter ≈3 mm, penetrated two dumbbell-shaped samples (1 mm thick, fixed into a frames), which sandwiched the source, and annihilate due to the thermalization process (Figure 1).

In our previous studies, the method for adjustment of the appropriate torque to fix a deformed sample in a frame to prevent/minimalize sample shrinkage and relaxation during PALS measurements was described in detail [49,52]. In a nutshell, the SAXS patterns for fixed samples with different torques were compared with the scattering patterns measured during in situ SAXS measurement by synchrotron beamline for material deformed to the same LS. The similarity of the scattering patterns collected for pseudo-in situ and in situ modes was an indicator of the correct value of torque. For HDPE deformed at a strain rate of 3.3 × 10^−3^ s^−1^, the estimated screw-tightening torque value was 5.6 in.-lb [49]. However, due to the absence of SAXS data from in situ measurements for the deformation conditions applied in this work, a different adjustment method was validated and implemented. We calculated the LS of the sample, fixed in a tensile testing machine, using sputtered dots and compared it to the LS value measured after fixing the sample in a frame with various torques. Undeformed sample and sample fixed in the frame were photographed on millimeter paper to allow adjustment for the scale of both pictures and to calculate LS (Figure S1). Additionally, samples in the frames were analyzed using SAXS to determine the intensity of scattering patterns or the value of the long period (for samples deforming in non-cavitating manner). For those values of torques that provided comparable LS to values registered straight after the deformation process in a tensile testing machine, an analysis of LS and the intensity of scattering patterns or the value of the long period as a function of time was additionally performed to select the appropriate torque.

Firstly, the method was validated on HDPE stretched under standard conditions (3.3 × 10^−3^ s^−1^ at 20 °C, Figure S2). The results fall in line with those from the method presented in previous work [49], which proves that the analysis of LS and the intensity of scattering patterns obtained under pseudo-in situ conditions can be an effective method for determining the optimal torque value to prevent the sample relaxation process during PALS measurements. In Figures S3–S6, the results of adapting the torque value for HDPE under different strain rates and temperatures are presented. For a temperature of 20 °C, strain rates of 3.3 × 10^−2^ s^−1^ (Figure S3) and 3.3 × 10^−4^ s^−1^ (Figure S4), the appropriate screw-tightening torque values were 6.4 in.lb. and 5.4 in.lb., respectively. For a strain rate of 3.3 × 10^−3^ s^−1^ and temperatures of −30 °C (Figure S5) and 70 °C (Figure S6), the appropriate screw-tightening torque values were 7.2 in.lb. and 4.2 in.lb., respectively. In Figures S7 and S8, the results of adapting the torque value for LDPE under different strain rates and temperatures are presented. For a strain rate of 3.3 × 10^−3^ s^−1^/temperature of 20 °C (Figure S7) and a strain rate of 3.3 × 10^−2^ s^−1^/temperature of −30 °C (Figure S8), the appropriate screw-tightening torque values were 4.8 in.lb. and 5.0 in.lb., respectively.

The exposure for the radiation took 48 h and was correlated with total number of counts in the spectrum (which should be around 2 × 10^6^ counts). The data collected during the PALS measurements were processed with use of LT-9.0 software [60]. The spectra were described by three components, τ_1_, τ_2_ and, τ_3_; three corresponding intensities, I_1_, I_2_ and I_3_; and three dispersions, σ_1_, σ_2_ and σ_3_. Component τ_1_ describes the shortest lifetime and it is approximately equal to the annihilation lifetime of p-Ps. τ_2_ corresponds to the free-positron annihilation, while τ_3_ corresponds to the pick-off annihilation, which occurs when o-Ps is trapped in a defect or micro-gap located within an amorphous regions.

It is assumed that free volume can be described as spherical pore with o-Ps in the center of it and electrons from adjacent molecules on the walls. Due to pick-off annihilation with an adjacent electron, o-Ps lifetime shortens to a few nanoseconds. Thus, o-Ps lifetime is directly correlated with the distance between o-Ps and the electrons of neighboring molecules [61,62]. Hence, the free-volume hole radius could be calculated from the o-Ps lifetime according to the Tao-Eldrup model [61,63]:

(2) $$\tau_{3}=\frac{1}{\lambda_{3}}=\frac{1}{2}{\left[1-\frac{r}{r+∆r}+\frac{1}{2\pi }sin\left(\frac{2\pi r}{r+∆r}\right)\right]}^{-1}\left[ns\right]$$

where Δr stands for an empirical constant correlated with the thickness of a homogeneous electron layer on a free-volume hole surface that amounts to 0.166 nm in molecular solids. Hence, the normalized γ(V) size distributions of free-volume pores detected by Ps in the analyzed polymers are able to be calculated with the equation:

(3) $$\gamma \left(V\right)=\frac{{-L}_{n}\left(\lambda_{3}\right)\frac{d\lambda_{3}}{dr}}{{4\pi r}^{2}}$$

where log-normal distributions L_n_(λ_3_) were calculated with the use of τ_3_ and σ_3_ values determined from LT-9.0 software, while the parameter dλ_3_/dr was calculated from Equation (2). The γ(V) distributions were normalized to the same area under the curve. More details about this methodology were presented elsewhere [49,52].

## 3. Results and Discussion

During the research, two different polymers were analyzed. HDPE and LDPE are both polymers synthesized from the same monomer, ethylene, but they exhibit different properties due to variations in the degree of branching. In Table 1, the structural parameters of the materials used in the studies are presented. The degree of crystallinity was calculated using the specific heat of fusion value for perfectly crystalline polyethylene, which is 293 J/g [64]. The different architectures of the macromolecules in LDPE and HDPE resulted in materials with significantly different degrees of crystallinity, amounting to 39% and 68%, respectively. Using the crystalline volume fraction (X_c_ (vol)) and the value of the long period (LP), the thickness of lamellar crystals and amorphous layers were also determined (Table 1). The thickness of crystals amounted to 4.2 nm and 13.4 nm for LDPE and HDPE, respectively. Meanwhile, the thickness of the amorphous layers was practically constant and was about 8 nm regardless of the type of polyethylene.

**Figure 2:**
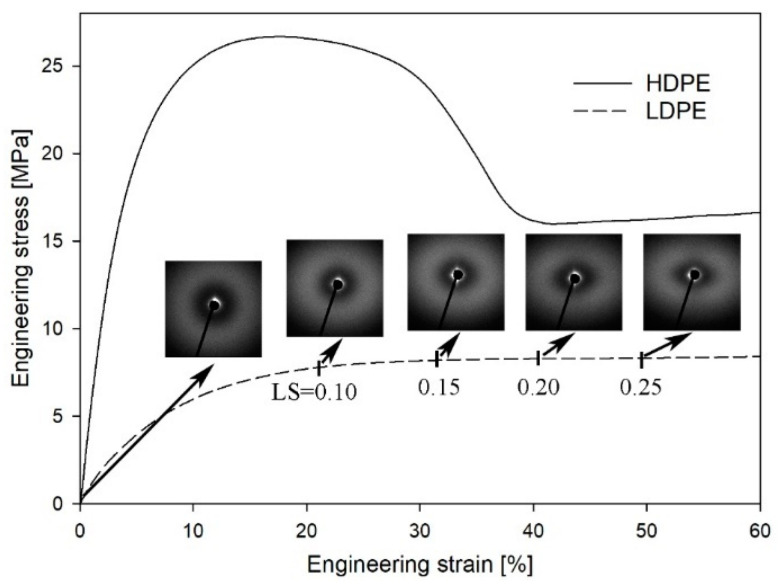
Engineering strain–engineering stress curves for HDPE and LDPE (temperature of 20 °C, strain rate of 3.3 × 10^−3^ s^−1^). Evolution of SAXS pattern for LDPE with the increase in the local strain value (LS). Direction of deformation: vertical.

According to their different morphologies, HDPE and LDPE are characterized by different mechanical properties. In Figure 2, the mechanical curves of both materials are presented. HDPE displayed a typical shear-yielding deformation with a microneck formation process. The macroscopic yield point was observed at a strain value of 20%, which corresponds to a LS within the range of 0.12 to 0.15. Additionally, in our previous work the mechanical curve was matched with SAXS patterns registered for different local strains [49]. Cavitation was observed in SAXS patterns by the appearance of a characteristic scattering signal within an LS range of 0.12–0.15 [49]. PALS analysis, conducted in the LS range 0–0.25, revealed that the mean ortho-positronium lifetime (τ_3_), corresponding to the average size of the free-volume pores in the amorphous regions, decreased in comparison with the undeformed material, even after the initiation of cavitation pores. This phenomenon was induced by the highly anisotropic, ellipsoidal shape of cavities, with the aspect ratio amounting to approximately 45. As a result, only the shortest axis of the pores was detectable using the PALS technique. It was also proven that the cavitation phenomenon was responsible for the increase in the dispersion of the ortho-positronium lifetime (σ_3_). Additionally, no changes in the mean positron lifetime (τ_2_) and a decrease in its dispersion (σ_2_) were observed. These effects were caused by mutually compensating changes of interplanar spacing within the crystalline component and by the relative displacement (slips) of crystalline blocks within individual lamellae, respectively. All details regarding this research (experimental data, discussion, conclusions) are included in a previous report [49].

**Figure 3:**
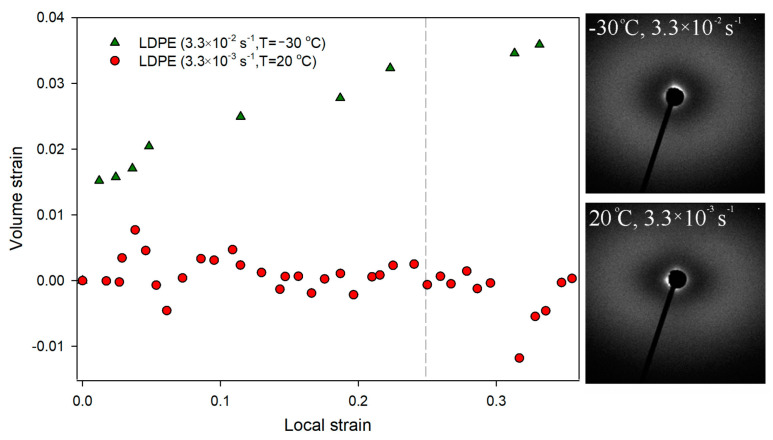
Dependence between volume strain and local strains for analyzed low-density polyethylene. The SAXS patterns for samples deformed to the local strains of 0.25 at −30 °C/3.3 × 10^−2^s^−1^ and at 20 °C/3.3 × 10^−3^s^−1^. Direction of deformation: vertical.

The deformation of LDPE occurs in a slightly different way than the deformation of HDPE; it occurs homogenously within gauge length, without a microneck-formation process (Figure 2). A macroscopic yield point was observed at a strain value of 30%, which corresponds to a LS of 0.15. It is well known that LDPE, under standard deformation conditions (3.3 × 10^−3^ s^−1^ at 20 °C), does not cavitate [66]. This effect results from the low plastic resistance of highly defective and thin LDPE crystals [67]. To confirm this, SAXS patterns were collected for selected LS values (Figure 2). No characteristic scattering signals were observed close to the beam stop, clearly confirming that within the range of LS values 0–0.25 the analyzed LDPE did not cavitate. The lack of cavitation process during uniaxial stretching of LDPE was also clearly visualized with the use of volume strain measurements. Figure 3 shows no changes in the volume strain, which proves that the deformation proceeds in a non-cavitating manner.

**Figure 4:**
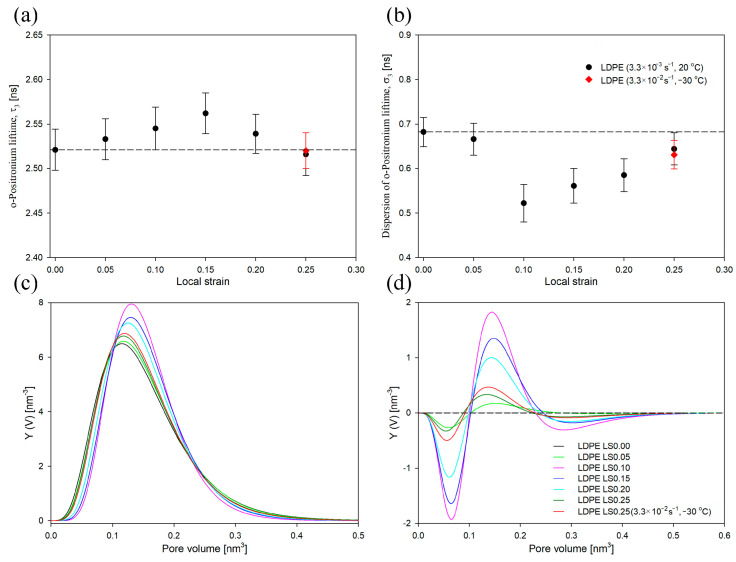
Mean ortho-positronium lifetime (τ_3_) (**a**) and dispersion of ortho-positronium lifetime (σ_3_) (**b**) for low-density polyethylene as a function of LS. The dashed lines represent the value for the undeformed sample. Normalized size distributions of free-volume pores of the amorphous phase of non-deformed LDPE and of samples deformed to selected local strains: as collected (**c**) and after subtraction of the distribution for the non-deformed sample (**d**). Deformation conditions were as follows: strain rate of 3.3 × 10^−3^ s^−1^/temperature of 20 °C and strain rate of 3.3 × 10^−2^ s^−1^/temperature of −30 °C.

It is also worth mentioning that the deformation of low-density polyethylene has never been previously analyzed using the PALS technique. Therefore, prior to analysis of the influence of strain rate and temperature on the deformation behavior of LDPE, PALS analysis was performed on this material that was deformed under standard deformation conditions (strain rate of 3.3 × 10^−3^ s^−1^/temperature of 20 °C) within the range of LS values 0–0.25. In Figure 4, the mean ortho-positronium lifetime (τ_3_) and its dispersion (σ_3_) as a function of LS values are presented. Additionally, for a better visualization of PALS data, the size distributions of free-volume pores for deformed samples, both as collected and after subtracting the distribution for undeformed LDPE, are shown (Figure 4c,d)

As depicted in Figure 4, both the mean ortho-positronium lifetime (τ_3_, 2.521 ns) and the dispersion of the ortho-positronium lifetime (σ_3_, 0.682 ns) for undeformed low-density polyethylene were noticeably higher compared to the high density polyethylene analyzed in our previous study: 2.384 ns and 0.499 ns, respectively [49]. This meant that both the average size of the free-volume pores of the amorphous phase and their size distribution were clearly larger in LDPE. This effect was probably induced by a lower molecular-packing density within the amorphous regions, resulting from the higher density of branching in low-density polyethylene. During stretching, the value of τ_3_ initially increased gradually up to the yield point (LS = 0.15), then it decreased slightly for a LS of 0.25 but remained close to the value observed in the undeformed sample. Thus, within the studied range of strains, the average size of the free-volume pores in the amorphous phase was generally higher than that in the undeformed sample. Analogous conclusions can be drawn from the evolution of size-distribution profiles, which generally shift towards higher values (Figure 4c).

On the other hand, as mentioned above, in the case of the previously analyzed HDPE, the mean ortho-positronium lifetime (τ_3_) was decreased compared to undeformed HDPE [49]. This difference can be correlated with the different mechanical response of the amorphous regions of these polyethylenes. It is well known that, in the case of lamellar crystals oriented parallel to the deformation direction, the amorphous component located between them undergoes compression, resulting in a decrease in the mean size of free-volume pores (decrease in τ_3_). Meanwhile, the amorphous component located between the lamellae oriented perpendicular to the deformation direction undergoes stretching as a result of the lamellae-separation process, which is manifested by an increase in the mean size of free-volume pores (increase in τ_3_). In the case of HDPE at a LS of 0.15, the change in thickness of amorphous regions between lamellae oriented parallel and perpendicular to the deformation directions was −4.8% and 17.5%, respectively [49]. In the case of LDPE at a LS of 0.15, the change in thickness of the amorphous regions between lamellae oriented parallel and perpendicular to the deformation directions, estimated based on the initial thickness of the amorphous regions (Table 1) and observed changes in LP (Figure S9), was −10.1% and 31.8%, respectively The relative increase in the content of interlamellar spaces during the deformation of LDPE was therefore clearly higher than in HDPE. This would explain the observed differences in the values of τ_3_ in a function of LS between LDPE and HDPE. It is worth mentioning that the observed differences in the deformability of amorphous regions of LDPE and HDPE can be attributed to the differences in the stiffness of this component. Recently, we demonstrated that the elastic modulus of the amorphous regions of LDPE is significantly lower than that in HDPE: ≈4 MPa and ≈40 MPa, respectively [12,13].

The dispersion of ortho-positronium lifetime (σ_3_, Figure 4b) reached a minimum at a local strain of 0.1, then gradually increased for higher LS values. This effect was mainly caused by a significant reduction in the content of free volume pores of the smallest and, to some extent, the largest sizes (Figure 4c,d). It is worth mentioning that a significant increase in the value of σ_3_ for HDPE (compared to the undeformed sample) was an indicator of the cavitation phenomenon [49]. In the case of LDPE at a LS of 0.25, this parameter remained below the value characteristic for the undeformed sample, which is consistent with the above conclusions that this polymer does not cavitate.

### 3.1. Strain Rate

**Figure 5:**
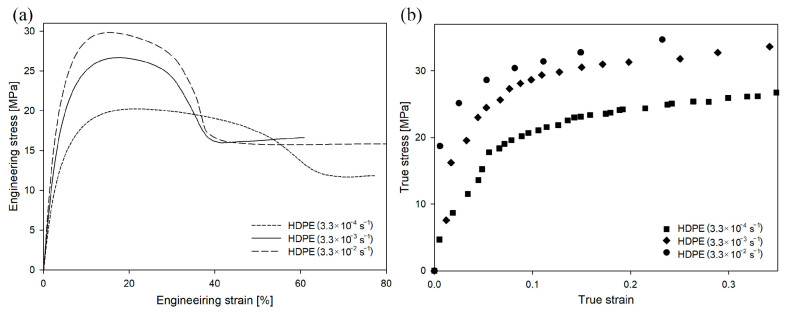
Engineering strain–engineering stress (**a**) and true strain–true stress (**b**) curves of HDPE as a function of strain rate at a constant temperature of 20 °C.

When the same polymer is stretched under different conditions, the engineering/true strain–engineering/true stress curves change shape. In the case of HDPE deformed at different strain rates (Figure 5), the value of engineering or true stress increased with an increasing deformation rate. This trend was visible in the early stages of the deformation process, but the difference in stress values became particularly significant near the yield point. The yield stress for the drawing rate of 3.3 × 10^−4^ s^−1^ was 22.5 MPa (yield-stress values in this study were determined from the true stress–true strain curves), for the drawing rate of 3.3 × 10^−3^ s^−1^ was 29.2 MPa, and for the drawing rate of 3.3 × 10^−2^ s^−1^ was 30.5 MPa. A similar influence of strain rate on yield stress has been observed by others [68].

**Figure 6:**
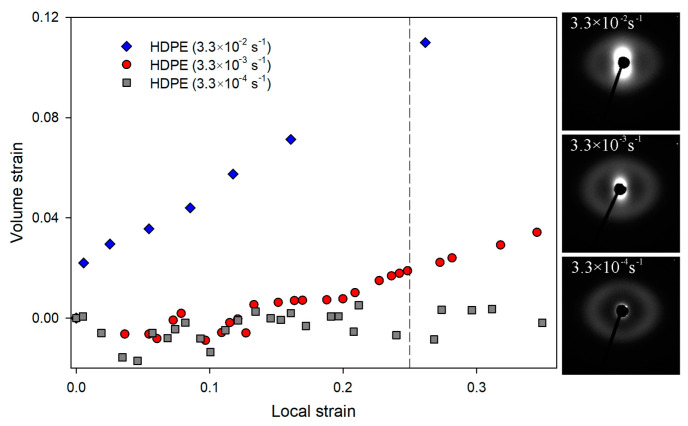
Dependence between volume strain and LS for HDPE as a function of strain rates, and the SAXS patterns for samples deformed to the LS of 0.25. Direction of deformation: vertical.

It is well known that the strain rate influences the intensity of the cavitation phenomenon [43,69,70]. As shown in Figure 6, HDPE drawn with a strain rate of 3.3 × 10^−2^ s^−1^ showed the highest increase in volume strain values since the very beginning of the deformation process. At a LS of 0.25, the volume strain was approximately five times higher than that of the sample deformed with a strain rate of 3.3 × 10^−3^ s^−1^. A measurable difference between samples deformed at strain rates of 3.3 × 10^−3^ s^−1^ and 3.3 × 10^−4^ s^−1^ appeared around the LS value of 0.13. This LS value was estimated in our previous work as the deformation stage at which the cavitation phenomenon was initiated (for a strain rate of 3.3 × 10^−3^ s^−1^) [49]. In the case of HDPE stretched at the lowest strain rate (3.3 × 10^−4^ s^−1^), no increase in volume strain was observed, which clearly indicates a complete inhibition of cavitation. SAXS patterns collected for samples deformed to a LS of 0.25 confirmed the influence of strain rate on the intensity of the cavitation process described above (Figure 6).

**Figure 7:**
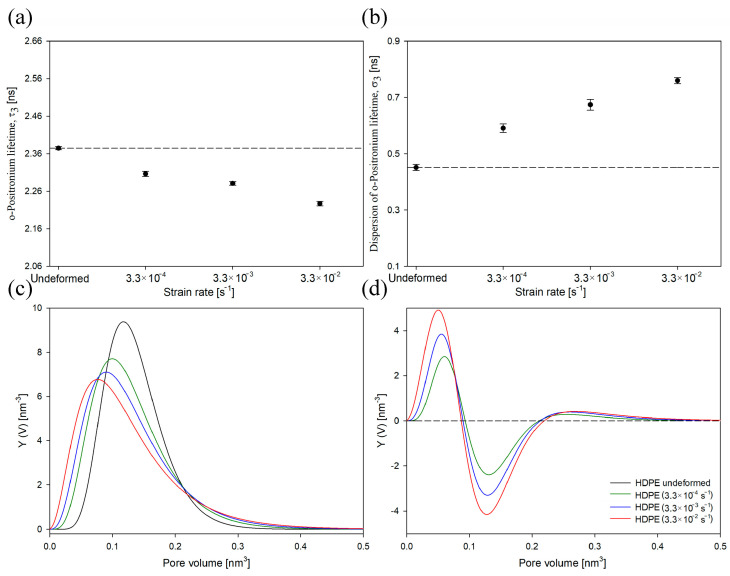
Mean ortho-positronium lifetime (τ_3_) (**a**) and dispersion of ortho-positronium lifetime (σ_3_) (**b**) for HDPE at a local strain of 0.25 as a function of strain rate. The dashed lines represent the value for undeformed samples. Normalized size distributions of free-volume pores in the amorphous phase of non-deformed HDPE and samples deformed with different strain rate values at a LS of 0.25: as collected (**c**) and after subtraction of the distribution for the non-deformed sample (**d**).

In Figure 7a,b, the mean ortho-positronium lifetime (τ_3_) and its dispersion (σ_3_) for undeformed HDPE and samples deformed to the LS of 0.25 with different strain rates were presented, respectively. Additionally, for a better visualization of PALS data, the size distributions of free-volume pores for deformed samples, both as collected and after subtracting the distribution for undeformed HDPE, are presented (Figure 7c,d).

As shown in Figure 7a, τ_3_ decreased gradually as the strain rate increased. This meant that the average size of the free-volume pores of the amorphous phase also decreased. Considering the observed increase in cavitation intensity with a higher strain rate (Figure 6), this effect was rather surprising. One would expect the opposite trend. However, this phenomenon can be explained by considering the initial highly anisotropic/ellipsoidal shape of the cavities. As mentioned above and detailed in our previous studies [49,52], only the shortest axis of the cavities is detectable using the PALS technique. It should be considered that the initial thickness of the amorphous layers in all analyzed systems was identical. It is also known that the thickness of the amorphous layer naturally limits the size of the cavitation pores along the short axis. Therefore, with an increase in the strain rate/cavitation intensity, the number or aspect ratio of cavitation pores can primarily increase, whereas the short pore axis will not change significantly. Consequently, only a slight increase in the content of large-sized cavities (range of pore volume of 0.2–0.4 nm^3^, Figure 7c,d) with an increase in the strain rate/cavitation intensity was observed. At the same time, with the increase in strain rate, a clear increase in the content of pores with the smallest sizes was observed (range of pore volume of 0–0.1 nm^3^, Figure 7c,d). This process most likely takes place between the lamellar crystals whose deformation is accompanied by compression of the amorphous layers. This, in turn, ultimately led to a decrease in the τ_3_ value, as depicted in Figure 7a. With an increase in the strain rate/cavitation intensity, the dispersion of the mean ortho-positronium lifetime also increased (Figure 7b). The highest increase in σ_3_ value was observed for sample deformed with the highest strain rate/cavitation intensity.

**Figure 8:**
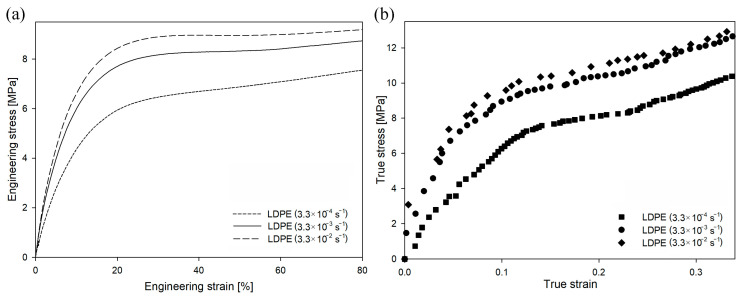
Engineering strain–engineering stress (**a**) and true strain–true stress (**b**) curves of LDPE as a function of strain rate at a constant temperature of 20 °C.

In the case of LDPE deformed at different strain rates (as shown in Figure 8), the value of engineering stress also increased with an increasing deformation rate. However, the changes in yield stress were lower than those in HDPE. The yield stress for the drawing rate of 3.3 × 10^−4^ s^−1^ was 7.6 MPa, for the drawing rate of 3.3 × 10^−3^ s^−1^ was 9.4 MPa, and for the drawing rate of 3.3 × 10^−2^ s^−1^ was 10.1 MPa.

In the case of HDPE, the stress at the yield point had to exceed 22–29 MPa before the cavitation process could be initiated (Figure 5 and Figure 6). Assuming that the susceptibility of the amorphous phase of LDPE and HDPE to a loss of consistency and to the formation of cavities during deformation is similar, in order to observe cavitation during deformation of LDPE, an analogous stress at the yield point should be obtained. Meanwhile, for LDPE, even when the highest strain rate was used, the yield-stress value was relatively low. Therefore, it was expected that no cavitation pores would develop during the deformation of LDPE under the given deformation conditions. Instead, plastic deformation of the crystals would be initiated before the stress exceeded the critical mechanical resistance of amorphous regions. The volume strain measurements presented in Figure S10 confirmed this hypothesis. Within the analyzed range of strain rates, an increase in volume strain as a function of LS was not observed, so the LDPE samples deformed without cavitation. PALS analysis (not presented here) did not reveal significant differences in the structural responses of LDPE samples (compared to HDPE samples) deformed in the analyzed range of strain rates.

### 3.2. Temperature

**Figure 9:**
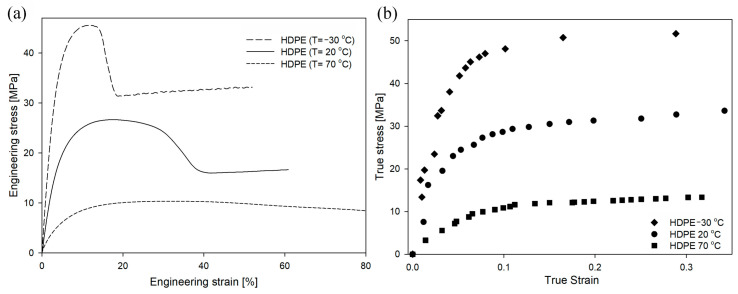
Engineering strain–engineering stress (**a**) and true strain–true stress (**b**) curves of HDPE as a function of temperature, at a constant strain rate of 3.3 × 10^−3^ s^−1^.

Changes in the temperature of the deformation process had a significant impact on the tensile behavior of the analyzed polymers. In the case of HDPE (Figure 9), decreasing the temperature to −30 °C resulted in an increase in stress value from the very beginning of the process and the appearance of the yield point at a lower strain value compared to standard strain conditions. Consequently, it was impossible to prepare a homogenously deformed sample with a LS of 0.25, as was the case with the other samples. PALS measurements were therefore performed for samples deformed at −30 °C to a maximum local strain value of 0.17. On the contrary, increasing the deformation temperature to 70 °C resulted in a decrease in stress values across the range of studied strains and practically suppressed the micronecking process typically observed in the curves of HDPE deformed at temperatures of −30 °C and 20 °C (Figure 9). The yield stress for the temperature of −30 °C was 49.0 MPa, for the temperature of 20 °C was 29.2 MPa, and for the temperature of 70 °C was 11.5 MPa. Similar influences of temperature on yield stress have been observed by others [23,71,72].

**Figure 10:**
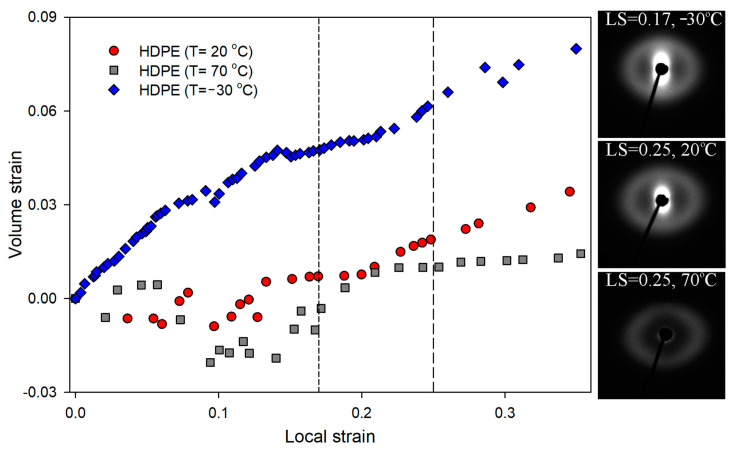
Dependence between volume strain and local strains for HDPE as a function of temperature. The SAXS patterns for samples deformed to a LS of 0.17 (−30 °C) and 0.25 (20 °C and 70 °C). Direction of deformation: vertical.

It has been shown in the literature that temperature influences the intensity of the cavitation phenomenon [38,72]. The analysis of the volume strain of HDPE, the results of which are presented in Figure 10, clearly shows that the volume strain depends on the temperature. The greatest increase in the value of volume strains was observed in HDPE drawn at the lowest temperature tested, i.e., −30 °C. At a LS of 0.17, the increase in volume of this sample was 5%. At this stage of deformation, at higher temperatures no change in volume strain was observed. At a LS of 0.25, the volume strain was approximately three times higher than that of the sample deformed at 20 °C. Meanwhile, in the HDPE sample stretched at the highest temperature (70 °C), no increase in volume strain was observed across the range of studied strains, indicating complete suppression of cavitation. SAXS patterns collected for samples deformed to local strains of 0.17 and 0.25 confirmed the influence of temperature on the formation of the cavities discussed above (Figure 10).

**Figure 11:**
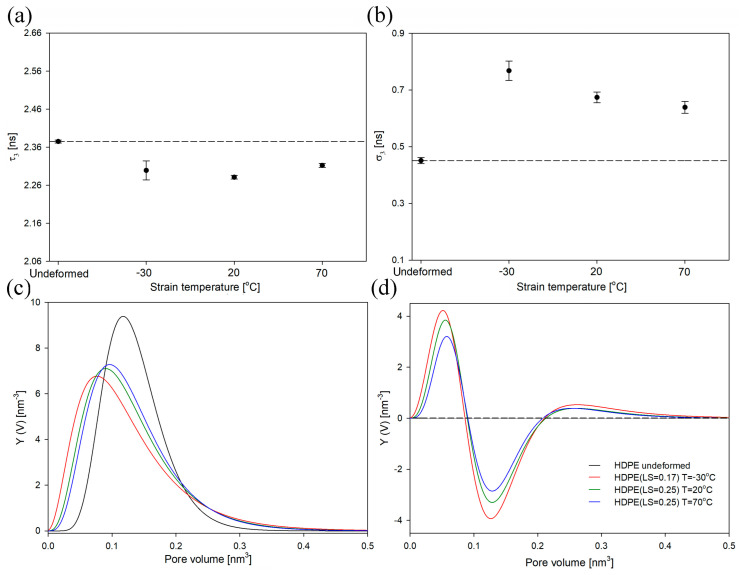
Mean ortho-positronium lifetime (τ_3_) (**a**) and dispersion of ortho-positronium lifetime (σ_3_) (**b**) for HDPE at a LS of 0.17 (−30 °C) and a LS of 0.25 (20 °C and 70 °C). The dashed lines represent the value for undeformed samples. Normalized size distributions of free-volume pores of the amorphous phase of non-deformed HDPE and samples deformed at different temperature values: as collected (**c**) and after subtraction of the distribution for the non-deformed sample (**d**).

In Figure 11a,b, the mean ortho-positronium lifetime (τ_3_) and its dispersion (σ_3_) for undeformed HDPE and samples deformed at different temperatures were presented. The PALS results for selected local-strain values are presented (0.17 and 0.25). The size distributions of free-volume pores for deformed samples, both as collected and after subtracting the distribution for undeformed HDPE, are also shown (Figure 11c,d).

The sample deformed at 70 °C exhibited the lowest changes in τ_3_ and σ_3_ values compared to the undeformed material (Figure 11a,b). It can therefore be assumed that the changes in the nanostructure of the amorphous phase at the free-volume level, including the mean size of free-volume pores and their size distribution, were the smallest. The greatest changes in the structure of the material after lowering the deformation temperature were observed in the size distribution of free-volume pores. This effect was mainly caused by a significant increase in the content of free volumes pores, particularly those of the smallest sizes and, to some extent, the largest sizes (Figure 11c,d). Similar effect was observed in HDPE after increasing the strain rate (Figure 7c,d). An insignificant increase in the content of large-sized cavities (in the range of pore volume of 0.2–0.4 nm^3^, as shown in Figure 11c,d) in the samples deformed at a lower temperature (which exhibited a higher intensity of cavitation, Figure 11) was explained earlier and is attributed to the initial highly anisotropic/ellipsoidal shape of cavities. The annihilation of the o-Ps along the longer axis of the cavities was highly unlikely, and this component was not present in the PAL spectrum. At the same time, the shorter axis of the cavities was naturally limited by the thickness of the amorphous layers; hence, despite a clear increase in cavitation intensity with a decrease in the deformation temperature, the rise in the content of large-sized cavities detectable by PALS was relatively low.

**Figure 12:**
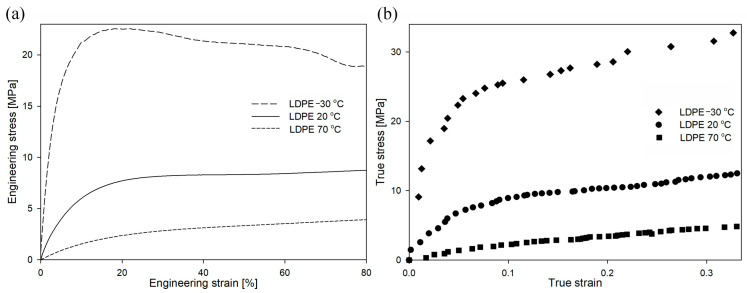
Engineering strain–engineering stress (**a**) and true strain–true stress (**b**) curves of LDPE as a function of temperature, at a constant strain rate of 3.3 × 10^−3^ s^−1^.

The mechanical curves for LDPE deformed at different temperatures are presented in Figure 12. The yield stress for the temperature of −30 °C was 25.0 MPa, for the temperature of 20 °C was 9.4 MPa, and for the temperature of 70 °C was 3.3 MPa. In the case of the sample deformed at −30 °C, the engineering curve clearly showed the micronecking phenomenon, previously exclusively observed in HDPE samples. It is also worth mentioning that the yield stress for this deformation condition was only slightly below the yield stress of the cavitating HDPE sample (Figure 5 or Figure 9). It could therefore be expected that the deformation of the LDPE sample at −30 °C would be accompanied by the phenomenon of cavitation. However, the volume strain measurements presented in Figure S11 did not confirm this hypothesis. Across the analyzed range of deformations, there was no increase in volume strain as a function of local strain, regardless of the deformation temperature. Therefore, the LDPE samples deformed within the analyzed range of temperatures/LSs without experiencing cavitation. The measurements with the use of PALS technique (not presented here) did not reveal significant differences in the structural changes of LDPE samples (in comparison to HDPE) deformed in the analyzed range of temperatures [49].

**Figure 13:**
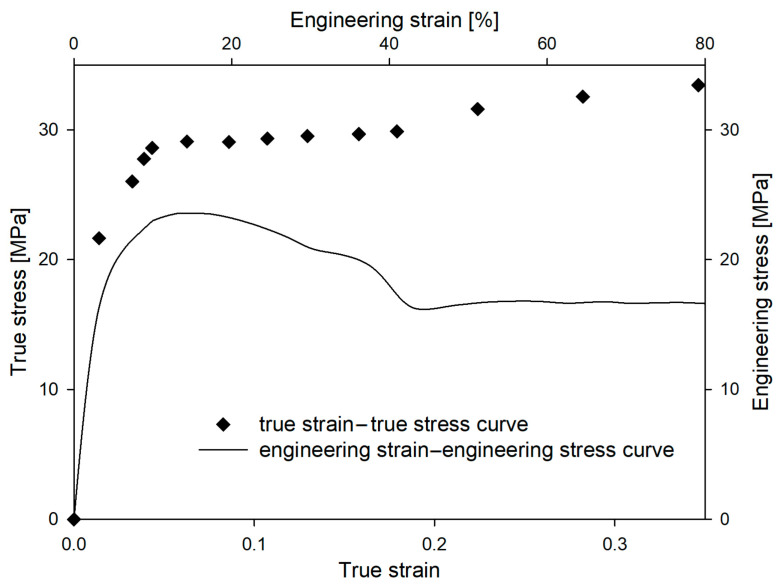
Engineering strain–engineering stress and true strain–true stress curves of LDPE deformed at −30 °C with a strain rate of 3.3 × 10^−2^ s^−1^.

In order to further “intensify” the mechanical response of LDPE, a deformation process was carried out at a temperature of −30 °C, increasing additionally the strain rate to 3.3 × 10^−2^ s^−1^. The mechanical curves for LDPE deformed at those conditions are presented in Figure 13. After the increase in strain rate, the process of deformation localization (microneck formation) observed in the engineering strain–engineering stress curve become even more distinguishable. In Figure 3 volume strain measurements for this LDPE sample are presented. With the increase in LS, a gradual increase in volume strain was observed from the earliest stages of the deformation process. At a LS of 0.25, the increase in sample volume was 3%. Such an effect usually indicates the formation of cavitation pores in the material. However, SAXS measurements did not confirm that cavitation was responsible for the observed increase in sample volume. No characteristic scattering from cavitation pores was observed in the SAXS pattern (Figure 3). This aspect will require further analysis.

It is worth mentioning that with the increase in the strain rate, the yield stress increased from 25 MPa to 29.1 MPa. Therefore, for the LDPE sample deformed at −30 °C with a strain rate of 3.3 × 10^−2^ s^−1^, the yield stress corresponded to that recorded for the cavitating HDPE deformed at 20 °C with a strain rate of 3.3 × 10^−3^ s^−1^. Meanwhile, as mentioned above, the LDPE sample did not cavitate. Therefore, the susceptibility of the amorphous phase of LDPE to lose its consistency and the formation of cavities is lower than in the case of amorphous component of HDPE. This can be correlated to the higher content of branches in LDPE, which ensures higher cohesion of the amorphous component.

In Figure 4a,b, the mean ortho-positronium lifetime (τ_3_) and its dispersion (σ_3_) for LDPE sample deformed at −30 °C with a strain rate of 3.3 × 10^−2^ s^−1^ for LS of 0.25 are presented, respectively (marked in red). Additionally, the size distribution of free-volume pores is shown (Figure 4c,d, red curve). Both τ_3_ and σ_3_ values were similar to the values recorded for the LDPE sample deformed at 20 °C with a strain rate of 3.3 × 10^−3^ s^−1^. Also, the size distribution profiles were very similar. Consequently, at a LS of 0.25, the nanostructure of the amorphous component of LDPE, including the mean size of free-volume pores and their size distribution, was similar regardless of the deformation conditions. It can therefore be concluded that the deformation conditions have limited influence on the evolution of structural changes in the amorphous component of LDPE under the range of strains studied.

## 4. Conclusions

HDPE and LDPE samples, solidified under the same conditions, were characterized by different nanostructure of amorphous regions. Both the mean ortho-positronium lifetime and the dispersion of ortho-positronium lifetime, corresponding to the average size and size distribution of the free-volume pores of the amorphous component, respectively, were noticeably higher in LDPE than in HDPE. This effect was induced by a lower and less uniform molecular packing within the amorphous regions, which resulted from the higher density of branching in low-density polyethylene.

During the deformation of LDPE, an increase in the τ_3_ value was observed under local strains within 0–0.25 compared to an undeformed sample. This effect was mainly stimulated by a significant increase in the interlamellar distances between lamellae oriented perpendicular to the deformation directions (31.8%), in contrast to a mere decrease (10.1%) in the interlamellar distances between lamellae oriented parallel to the deformation directions. No significant effect of temperature or strain rate on the τ_3_ value was observed during LDPE deformation.

Within the range of strains studied, the σ_3_ value of LDPE, and thus the size distribution of the free-volume pores within the amorphous component, generally remained below the value observed for the undeformed sample. This effect was mainly caused by a considerable reduction in the content of the smallest and, to some extent, the largest pores of free volumes. Consequently, the dimensions of free-volume pores became more uniform. No significant effect of temperature and strain rate on the σ_3_ value was observed during LDPE deformation.

A significant impact of the deformation condition on the evolution of the amorphous-phase nanostructure was observed in the case of HDPE. With an increase in the strain rate or a decrease in temperature, the cavitation phenomenon was intensified in HDPE. Meanwhile, a decrease in mean ortho-positronium lifetime was observed. This effect was mainly caused by a significant increase in the content of the smallest and, to some extent, the largest pores of free volumes. An insignificant increase in the content of large-sized pores (which were indeed cavities) in the samples deformed at a lower temperature or higher strain rate was attributed to the initial shape of the cavities. The annihilation of the o-Ps along the longer axis of the anisotropic/ellipsoidal cavities was highly unlikely. The input of this dimension into the PAL spectrum was therefore limited. At the same time, the increase in the dimension of the shorter axis of the cavities was limited by the thickness of amorphous layers; hence, the increase in the content of large-sized pores/cavities detectable by PALS was relatively low.

With an increase in the strain rate or a decrease in temperature, the dispersion of the mean ortho-positronium lifetime increased during the deformation of HDPE. The highest increase in the σ_3_ value was observed in the sample deformed at the highest strain rate or at the lowest temperature, and this effect was directly correlated with the initiation or intensification of the cavitation phenomenon.

The yield stress of the LDPE sample deformed at lowered temperature and increased strain rate was consistent with cavitating HDPE, but the LDPE sample did not cavitate. This meant that the susceptibility of the amorphous regions of LDPE to the formation of cavities is lower than that for the amorphous component of HDPE. This, in turn, can be correlated to the higher content of branches in LDPE, which ensures higher cohesion of the amorphous component.

## Figures and Tables

**Table 1:** Selected structural parameters of HDPE and LDPE.

	Crystalline Mass Fraction ^a^ X_c_ (w)	Crystalline Volume Fraction ^b^ X_c_ (vol)	Melting Temperature [°C] ^a^	Long Period (LP) [nm] ^c^	Thickness of Crystals (l_c_) [nm] ^d^	Thickness of Amorphous Layers (l_a_) [nm] ^d^
HDPE	0.68	0.64	132.4	20.9	13.4	7.5
LDPE	0.39	0.35	109.9	12.1	4.2	7.9

^a^ from DSC; ^b^ based on the crystalline mass fraction with use of densities of crystalline and amorphous components: d_c_ = 1.003 g/cm^3^, d_a_ = 0.850 g/cm^3^ [65]; ^c^ from SAXS; ^d^ from the value of long period and crystalline volume fraction.

## Data Availability

The authors confirm that the data supporting the findings of this study are available within the article.

## Supplementary Materials

The following supporting information can be downloaded at: https://www.mdpi.com/article/10.3390/polym16030420/s1. Figure S1: Visualization of measurement of LS recorded with use of sample fixed in tensile testing machine (a) and sample before stretching and after deformation fixed in the frame with specified torque (b). Figure S2: Values of local strain (LS) for HDPE samples mounted in frames with different torques for strain rate 3.3 × 10^−3^ s^−1^ at 20 °C. Figure S3: Values of scattering intensity and LS for HDPE samples mounted in frames with different torques for strain rate 3.3 × 10^−2^ s^−1^ at 20 °C. Figure S4: Values of scattering intensity and LS for HDPE samples mounted in frames with different torques for strain rate 3.3 × 10^−4^ s^−1^ at 20 °C. Figure S5: Values of scattering intensity and LS for HDPE samples mounted in frames with different torques for strain rate 3.3 × 10^−3^ s^−1^ at −30 °C. Figure S6: Values of scattering intensity and LS for HDPE samples mounted in frames with different torques for strain rate 3.3 × 10^−3^ s^−1^ at 70 °C. Figure S7: Values of scattering intensity and LS for HDPE samples mounted in frames with different torques for strain rate 3.3 × 10^−3^ s^−1^ at 20 °C. Figure S8: Values of scattering intensity and LS for HDPE samples mounted in frames with different torques for strain rate 3.3 × 10^−3^ s^−1^ at −30 °C. Figure S9: Long periods for LDPE determined from SAXS patterns as a function of local strain and orientation of lamellar crystals. Figure S10: Dependence between volume strain and local strains for LDPE as a function of strain rates. Figure S11: Dependence between volume strain and local strains for LDPE as a function of temperature.
