# Supplementary material for: Strain Rate and Temperature Influence on Micromechanisms of Plastic Deformation of Polyethylenes Investigated by Positron Annihilation Lifetime Spectroscopy
Source: Polymers (Basel). 2024 Feb 2;16(3):420. doi: 10.3390/polym16030420 (PMC10857360; doi:10.3390/polym16030420)
Supplement: Supplementary file 1 [file polymers-16-00420-s001.zip › polymers-2824930-supplementary.pdf]

Supporting Information

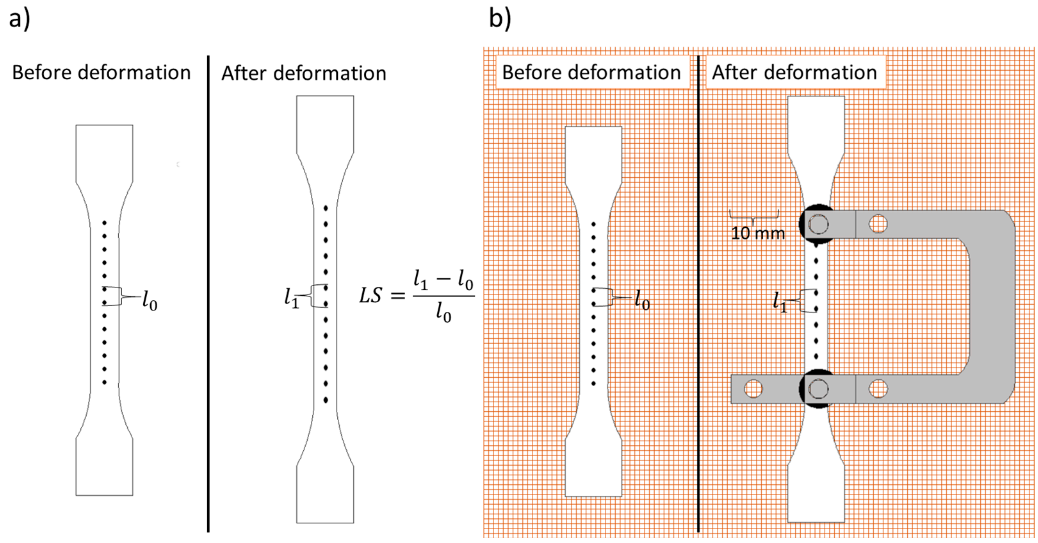

Figure S1. 2D Visualization of measurement of LS recorded with use of sample fixed in tensile testing machine (a) and sample before stretching and after deformation fixed in the frame with specified torque (b).

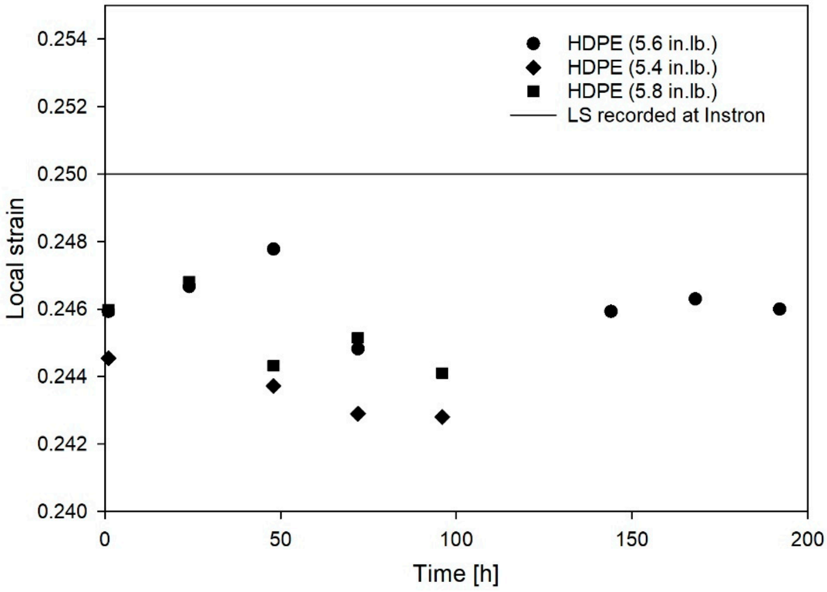

Figure S2. Values of local strain (LS) for HDPE samples mounted in frames with different torque for strain rate 3.3×10^{-3} s^{-1} at 20 °C.

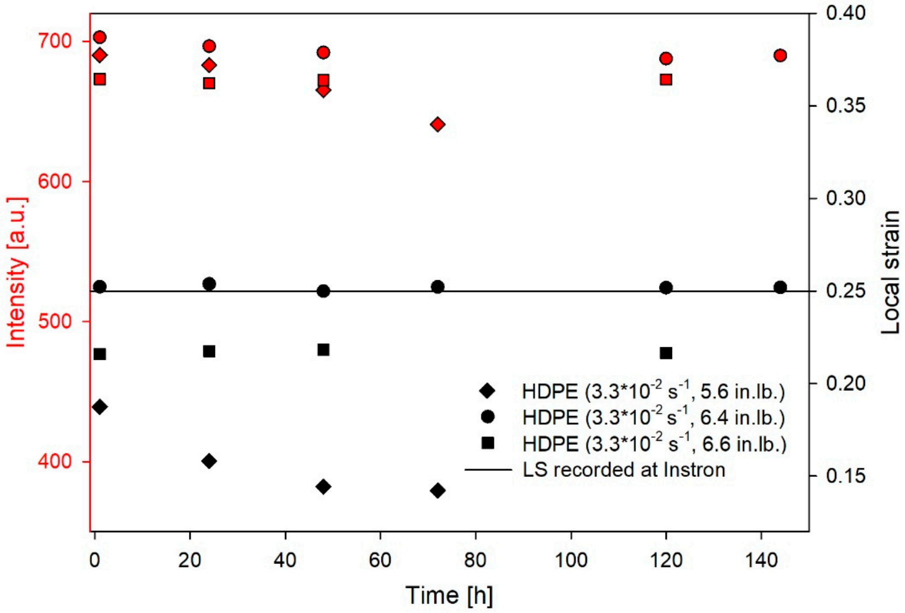

Figure S3. Values of scattering intensity and LS for HDPE samples mounted in frames with different torque for strain rate 3.3×10^{-2} s^{-1} at 20 °C.

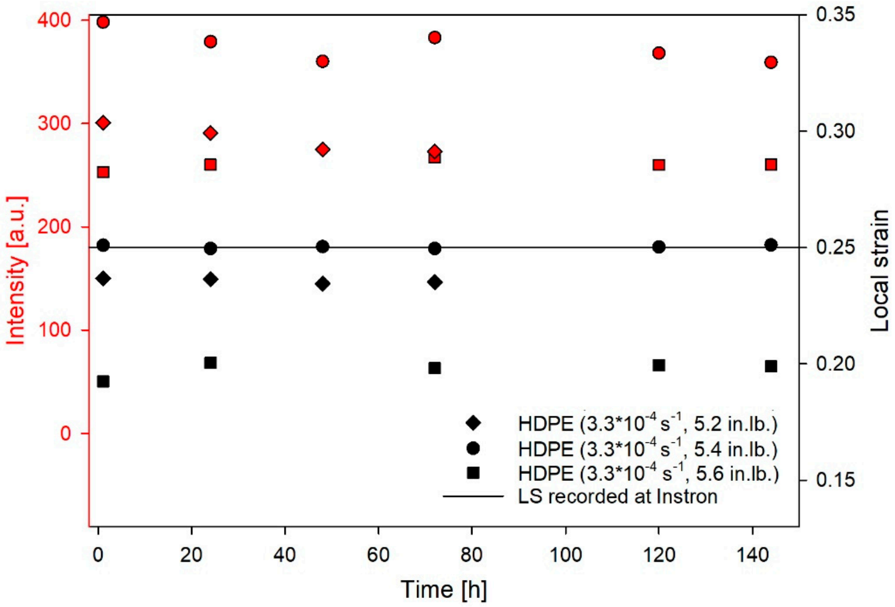

Figure S4. Values of scattering intensity and LS for HDPE samples mounted in frames with different torque for strain rate 3.3×10^{-4} s^{-1} at 20 °C.

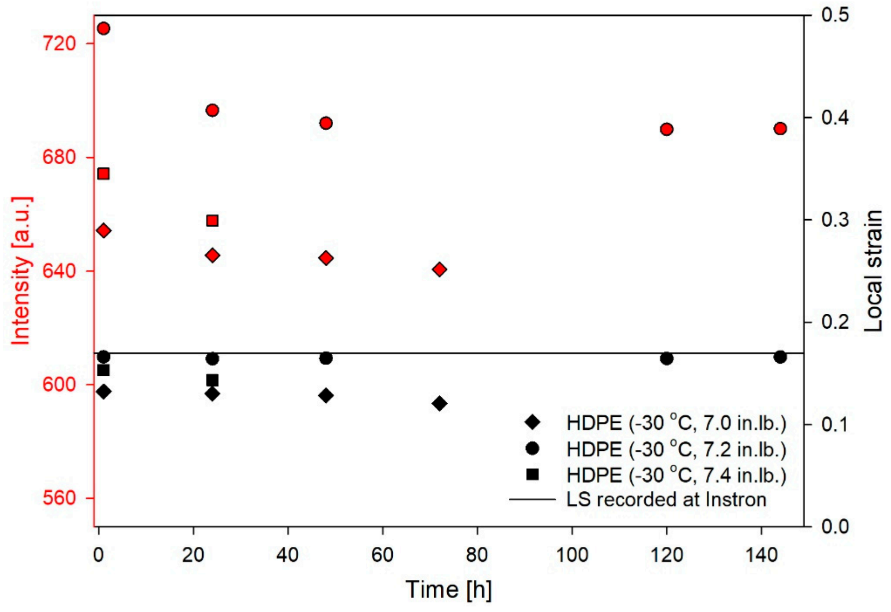

Figure S5. Values of scattering intensity and LS for HDPE samples mounted in frames with different torque for strain rate 3.3×10^{-3} s^{-1} at −30 °C.

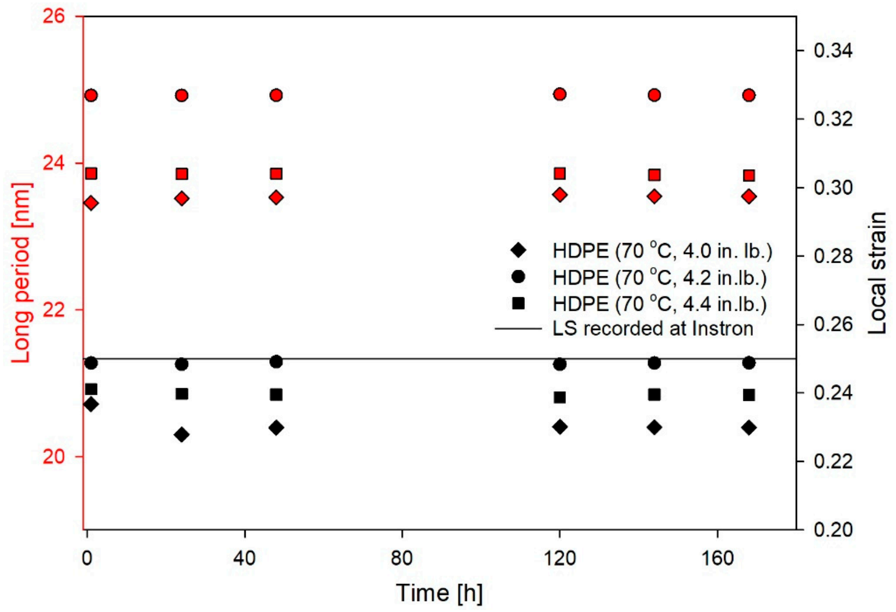

Figure S6. Values of scattering intensity and LS for HDPE samples mounted in frames with different torque for strain rate 3.3×10^{-3} s^{-1} at 70 °C.

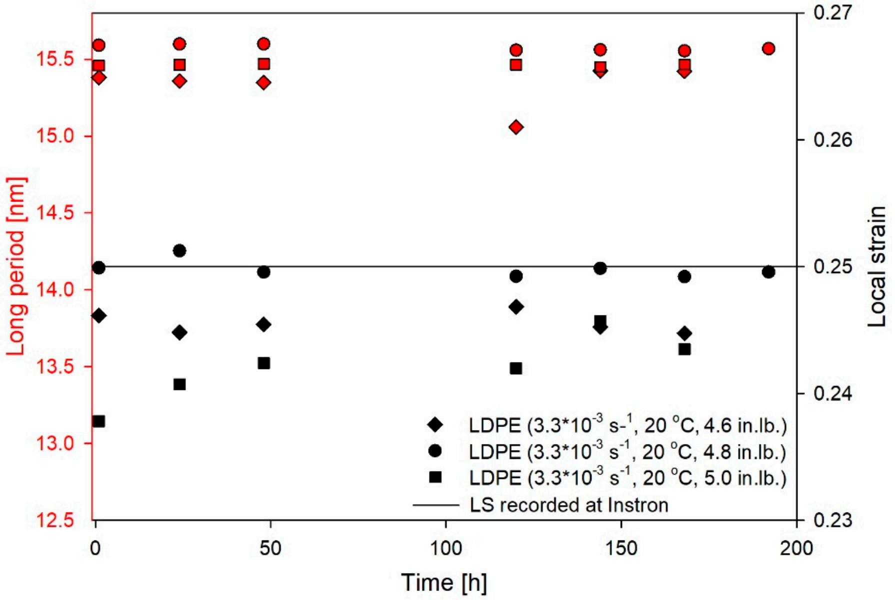

Figure S7. Values of scattering intensity and LS for HDPE samples mounted in frames with different torque for strain rate 3.3×10^{-3} s^{-1} at 20 °C.

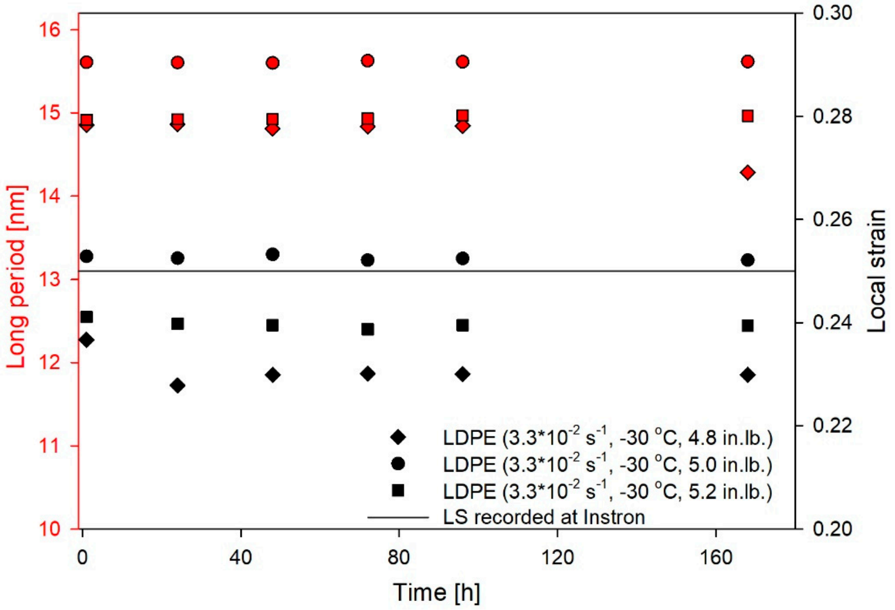

Figure S8. Values of scattering intensity and LS for HDPE samples mounted in frames with different torque for strain rate 3.3×10^{-3} s^{-1} at −30 °C.

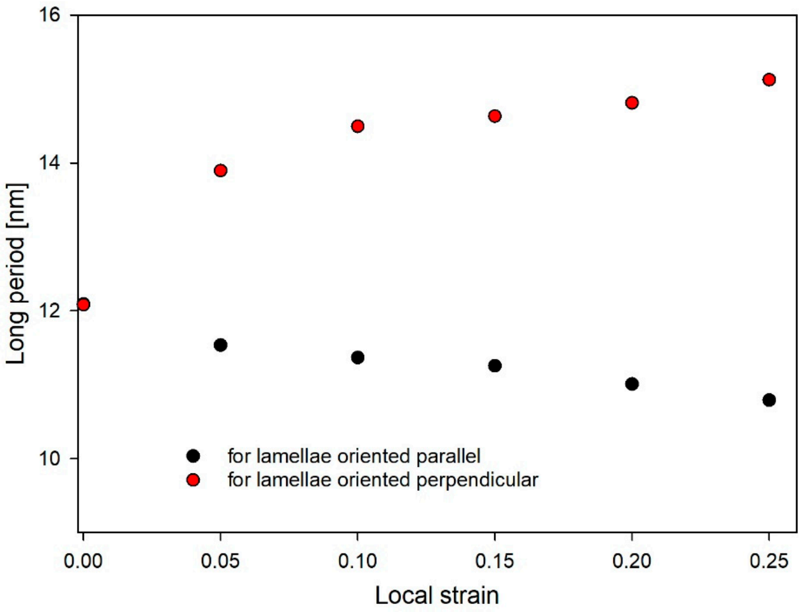

Figure S9. Long periods for LDPE determined from SAXS patterns as a function of local strain and orientation of lamellar crystals.

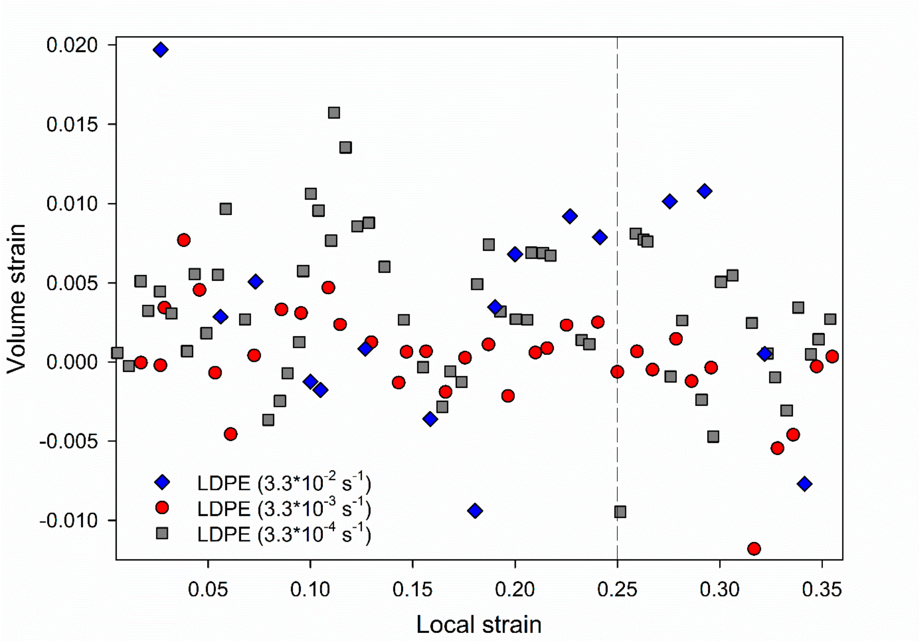

Figure S10. Dependence between volume strain and local strains for LDPE as a function of strain rates.

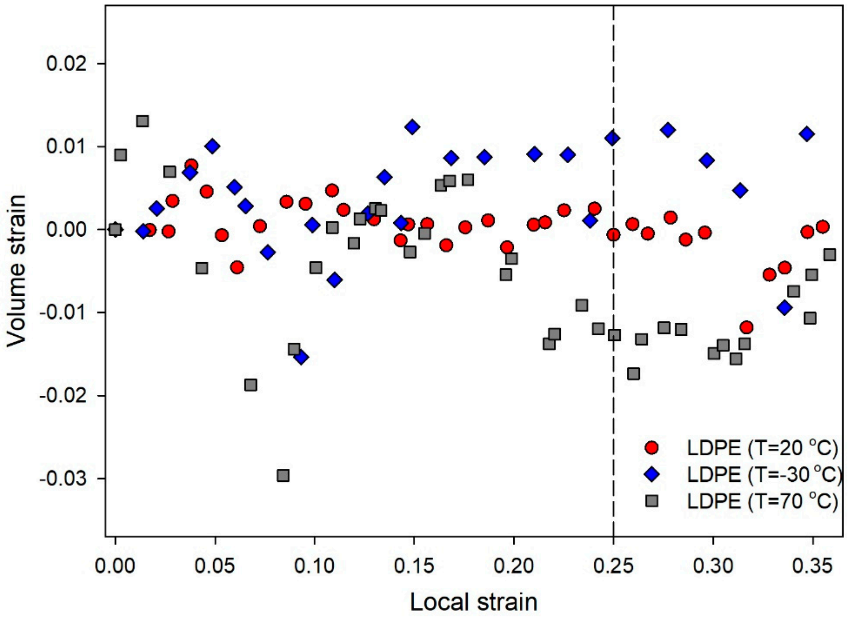

Figure S11. Dependence between volume strain and local strains for LDPE as a function of temperature.
